# Monocular Absolute Depth Estimation from Motion for Small Unmanned Aerial Vehicles by Geometry-Based Scale Recovery

**DOI:** 10.3390/s24144541

**Published:** 2024-07-13

**Authors:** Chuanqi Zhang, Xiangrui Weng, Yunfeng Cao, Meng Ding

**Affiliations:** 1 College of Astronautics, Nanjing University of Aeronautics and Astronautics, Nanjing 211106, China; chuanqi.zhang@nuaa.edu.cn (C.Z.); rickweng@nuaa.edu.cn (X.W.); 2 College of Civil Aviation, Nanjing University of Aeronautics and Astronautics, Nanjing 211106, China; nuaa_dm@nuaa.edu.cn

**Keywords:** monocular depth estimation, unmanned aerial vehicles, scale recovery, depth from motion, multi-view geometry

## Abstract

In recent years, there has been extensive research and application of unsupervised monocular depth estimation methods for intelligent vehicles. However, a major limitation of most existing approaches is their inability to predict absolute depth values in physical units, as they generally suffer from the scale problem. Furthermore, most research efforts have focused on ground vehicles, neglecting the potential application of these methods to unmanned aerial vehicles (UAVs). To address these gaps, this paper proposes a novel absolute depth estimation method specifically designed for flight scenes using a monocular vision sensor, in which a geometry-based scale recovery algorithm serves as a post-processing stage of relative depth estimation results with scale consistency. By exploiting the feature correspondence between successive images and using the pose data provided by equipped navigation sensors, the scale factor between relative and absolute scales is calculated according to a multi-view geometry model, and then absolute depth maps are generated by pixel-wise multiplication of relative depth maps with the scale factor. As a result, the unsupervised monocular depth estimation technology is extended from relative depth estimation in semi-structured scenes to absolute depth estimation in unstructured scenes. Experiments on the publicly available Mid-Air dataset and customized data demonstrate the effectiveness of our method in different cases and settings, as well as its robustness to navigation sensor noise. The proposed method only requires UAVs to be equipped with monocular camera and common navigation sensors, and the obtained absolute depth information can be directly used for downstream tasks, which is significant for this kind of vehicle that has rarely been explored in previous depth estimation studies.

## 1. Introduction

Monocular depth estimation (MDE) is an important research area in machine vision and vehicular sensing [1,2]. It aims to estimate a dense depth map from an input image. In recent years, this technology has received considerable attention and has been successfully applied in robotics [3] and autonomous driving [4]. Similarly, it is also of great value to other unmanned vehicles, particularly small unmanned aerial vehicles (UAVs) [5,6]. For small UAVs with limited sensors on board, the depth information of objects or the whole scene is crucial for various tasks, including environment perception [7] and obstacle avoidance [8].

The traditional MDE methods are mainly based on the multi-view geometry theory, which estimates depth by searching for motion cues or other information from image sequences captured by a monocular camera. A representative method is structure from motion (SfM) [9]. It employs handcrafted features to extract local information from the image, establishes feature matching relationships between images, and utilizes geometric constraints to calculate the depth. However, such methods can only produce sparse depth results, the accuracy of which depends on the image quality and the correctness of feature matching.

With the rapid development of deep learning and convolutional neural networks (CNNs), CNN-based MDE methods are constantly emerging, which can generate a dense depth map with the same resolution as the input image using an end-to-end architecture. These methods can be broadly categorized into supervised and unsupervised learning approaches, depending on whether the ground truth (GT) depth data are required during training [10,11]. In reality, it is very expensive to collect GT depth data on land, let alone in the air. Therefore, unsupervised MDE methods have a better prospect in practical applications of small UAVs.

**Figure 1:**
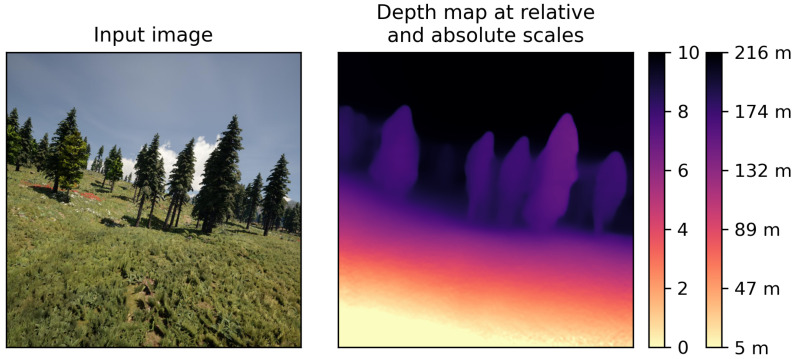
An example of relative and absolute depth estimation, where the visualization results are the same but the pixel values have different meanings. The right color bar with physical unit on ticks represents absolute depth, while the left bar represents relative depth.

Nevertheless, existing unsupervised MDE methods suffer from two fundamental problems that prevent them from being applied to flight scenes. The first is the scale problem, which arises from the inherent ambiguity of MDE [12]. Without the use of GT depth or stereo image pairs, no real-world scale information is introduced into the estimation process, so only relative depth maps can be generated, where the pixel values only represent the far and near relations between objects, but have no physical meaning. In absolute depth maps, however, the pixel values have a physical unit (such as meters), and indicate the real distance between points and the camera, as shown in Figure 1. Without the ability to obtain absolute depth, it can be quite difficult for UAVs to perform the tasks that rely on this kind of information, such as obstacle avoidance, autonomous landing and terrain following, and their flight safety may even be compromised.

The second problem is the poor adaptability of existing unsupervised MDE methods to UAV flight scenes, which are typically unstructured dynamic scenes. Most existing methods [13,14,15] are designed for semi-structured scenes, such as autonomous road driving, where the orientation of the vehicle changes only about one axis (i.e., the yaw angle). In such cases, the ground and sky are located at the top and bottom of an image, while the left and right sides are mostly geometric shapes such as curbs, road signs and building facades. Maximum depth often appears in the center of the image. This allows the CNN to learn the general structure of the scene. However, a UAV has 3 rotational degrees of freedom (DoF). With the presence of pitch and roll angles, the horizon and objects in images are tilted, the proportion of the sky and the ground does not remain unchanged and the geometric information is limited. All of these increase the difficulty for the CNN to learn scene structures and predict accurate depth maps. Without taking these factors into account, existing unsupervised MDE methods cannot be effectively applied to UAV flight scenes.

In this paper, we propose an absolute depth estimation method designed for small UAVs in flight. Absolute dense depth maps are generated from the image sequences captured by a monocular vision sensor and the navigation data (including the position and attitude of the UAV) provided by on-board navigation sensors such as a GPS receiver and an inertial measurement unit (IMU). Our method can be viewed as a post-processing of the relative depth predictions of unsupervised MDE, which only requires UAVs to be equipped with a monocular camera and common navigation sensors, without the need for other prior information and constraints. The main contributions of this paper are as follows:

- We propose a monocular absolute depth estimation method for UAV flight scenes. Our method extends the unsupervised monocular depth estimation technology from relative depth estimation in semi-structured ground scenes to absolute depth estimation in unstructured aerial scenes, which have not been as extensively studied. The multi-view geometry technology utilized in traditional MDE methods is introduced to address the problem of scale ambiguity faced by the modern unsupervised MDE approach.
- We propose a scale recovery algorithm based on multi-view geometry for disambiguation. A scale factor between relative and absolute scales is estimated for each frame, which allows the relative depth map to be multiplied pixel by pixel to obtain the corresponding absolute depth map. To achieve this, we take the displacement between adjacent frames as an intermediate quantity, the absolute value of which is available from the UAV positions, and the relative value of which can be calculated from the relative depth values of the matched points between two frames and the UAV navigation data. The quotient of the absolute and relative values of this quantity is taken as the scale factor.
- To make our method applicable to flight scenes, the attitude angles of the UAV obtained from its navigation sensors are introduced into the scale recovery algorithm to perform a series of geometric transformations, improving the accuracy of scale factors and absolute depth estimation results. Experiments also verify the robustness of our method to navigation sensor noise and its adaptability to different parameter settings, depth ranges and variations, and UAV motions.

This paper is organized as follows. Section 2 introduces related works on solving the scale problems in unsupervised MDE, including those that inspire us. Section 3 shows the pipeline of our monocular absolute depth estimation method and all the details. In Section 4, we select a part of the public aerial image dataset Mid-Air [16] to verify the effectiveness of our method, and provide quantitative and qualitative results to show its advantages. Finally, the conclusion is drawn in Section 5.

## 2. Related Work

### 2.1. CNN-Based MDE

Since 2012, the deep learning technology represented by CNN has achieved tremendous success in various vision tasks. In 2014, CNN was first applied to MDE by Eigen et al. [17]. At that time, MDE was regarded as a pixel-wise continuous regression problem. Let $I\in R^{w\times h\times 3}$ be a single RGB image with a resolution of w×h, and $D\in R^{w\times h}$ be the corresponding depth map. The CNN is trained to learn the mapping τ:I→D using a large training dataset containing images of similar scenes and corresponding GT depth maps, which is known as the supervised learning approach. With the development of CNN structure, the performance of supervised MDE methods has been continuously improved [18,19]. However, datasets with GT depth data of real scenes are expensive and scarce, and the generalization ability of these models in unknown scenes is also limited, making it difficult to achieve widespread application.

Unlike supervised methods, the unsupervised learning approach does not require GT depth during training. In contrast, the supervision signal for network training is derived from the geometric constraints between continuous images in a sequence [14], i.e., the target (current) image $I_{t}$ and a source image $I_{s}$. In 2016, Garg et al. [20] proposed the basic idea of unsupervised MDE, where the loss function is constructed by an image reconstruction process using the geometric relationship between two images. The target image is fed into CNN to predict the corresponding depth map $D_{t}$, which is then used to reconstruct a target image $I_{t}^{\prime }$ from $I_{s}$. And the loss is calculated based on the difference between the reconstructed and the original target images.

The idea of image reconstruction comes from the view synthesis process in multi-view stereo. For a pixel point in $I_{t}$, given its pixel coordinates $p_{t}$, its corresponding depth value $D_{t}\left(p_{t}\right)$ in the predicted $D_{t}$ and the camera’s intrinsic matrix K, then the projection of the corresponding 3D point P in the source image $I_{s}$ can be obtained by a series of transformations, as expressed by the following equations.

(1) $$p_{t}\overset{D_{t},\;K^{-1}}{\underset{\mathrm{Back}\;\mathrm{projection}}{\to }}P_{t}\overset{T_{t\to s}}{\underset{\mathrm{Transformation}}{\to }}P_{s}\overset{K}{\underset{\mathrm{Projection}}{\to }}p_{s}$$

(2) $$p_{s}∼KT_{t\to s}D_{t}\left(p_{t}\right)K^{-1}p_{t}$$

where $T_{t\to s}$ is the relative pose from the target view to the source view, and the use of equivalent symbol instead of equal sign is due to the loss of depth information of the 3D point during the final projection step. The whole process is implemented by the operation of inverse warping based on differentiable bilinear interpolation [21], which makes back propagation during network training feasible.

### 2.2. Scale Problems in Unsupervised MDE

**Figure 2:**
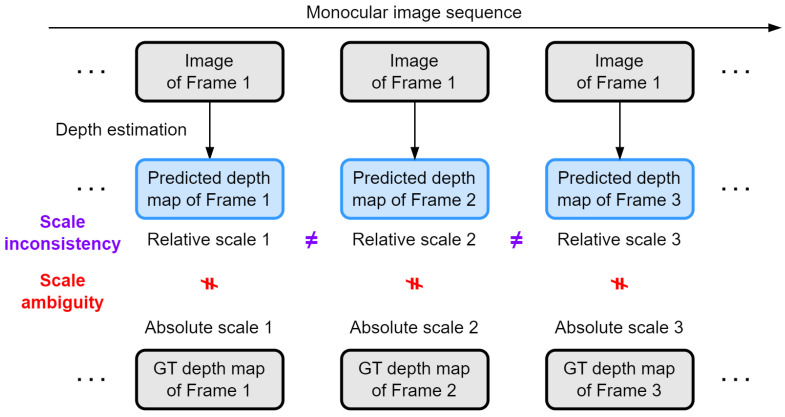
Illustration of the scale problems in unsupervised MDE.

Unsupervised MDE methods have relaxed requirements for training data, resulting in a wide range of applications and enormous potential. However, without GT depth as supervision, the scale of depth prediction can be different from that of the real world, which is called “scale ambiguity”. We cannot directly assign physical units such as meters to the pixel values in relative depth maps. Furthermore, the scale of predicted relative depth maps corresponding to images in a sequence may also be different, which is called “scale inconsistency”. For example, in an extreme case, for a given static point in 3D space, the predicted depth value in the current frame may be greater than that in a previous frame, which is completely unrealistic if the vehicle is moving forward. These two scale problems are illustrated in Figure 2, which undoubtedly hinder the practical application of unsupervised depth estimation methods.

Many studies on scale recovery have been conducted to deal with the ambiguity problem. In [22,23], the feature correspondence between current and previous images is used. By constructing line segments between feature points within the region of a certain object in the scene, the absolute depth of object can be calculated from the change in length of these segments and the horizontal displacement of camera between images. However, this kind of method only outputs depth values at the object level, not a dense depth map at the pixel level.

Beyond this, additional geometric constraints, such as the surface normal of buildings or the ground and the translation between frames, are used to recover the scale. For autonomous driving applications, Xue et al. [4] estimate the height of camera to the ground at the predicted relative scale using a dense geometric constraint introduced by ground pixels. Since the actual camera height is known, an accurate scale factor between relative and absolute scales can be calculated. This is an intuitive post-processing method from relative to absolute depth estimation, without the need to modify the existing depth estimation network. Petrovai et al. [24] use this method to produce pseudo labels for training a self-distillation framework and improve the accuracy. Unfortunately, the geometric constraints that exist in structured and semi-structured scenes (e.g., indoor scenes and driving scenes) are not common in unstructured UAV flight scenes. A more general approach is to use an auxiliary sensor. Zhang et al. [25] propose a scale-aware unsupervised MDE architecture by integrating the IMU motion dynamics. During training, the absolute scale is recovered from the IMU data by pre-integration, and then fused with the visual sensor information by a differentiable camera-centric extended Kalman filter (EKF). However, the simultaneous processing of data from both visual and IMU sensors complicates the network architecture. In addition to the relative depth and pose estimation networks, an EKF fusion module and two lightweight gravity and velocity estimation networks are also designed for IMU initialization as in the simultaneous localization and mapping (SLAM) task. The generalizability of this method has not yet been validated in UAV flight scenes.

For UAV applications, Pinard et al. [26] predicts absolute depth maps from a monocular video. The network architecture is similar to that of [14]. Both current and previous image frames are taken as input, and a frame stabilization step is applied during training to compensate the rotation between two frames. The translation component of the estimated pose is then normalized with a known fixed nominal displacement, which introduces the scale information of scene into the training process. However, this method is based on a strong assumption that the speed of vehicle is constant during flight, and the displacement between adjacent frames should be fixed before training, which is not always the case in practical applications.

Inspired by these works, we propose a method to solve the scale ambiguity problem in aerial scenes. We establish a relationship between relative and absolute scales via the physical quantity of the UAV’s displacement between frames, and calculate a plausible scale factor to transform relative depth predictions into absolute ones. Our method does not need to know the camera height as in [4], nor does it have the constant speed assumption as in [26], but only utilizes the position and attitude information provided by the navigation sensors commonly equipped on UAVs.

It should be emphasized that for this principle to work, the relative depth maps corresponding to the two images must be at the same scale, i.e., they are scale consistent. Fortunately, Bian et al. [27,28] present a scale-consistent MDE architecture by introducing a geometric consistency constraint between consecutive frames. The corresponding consistency loss is designed to minimize the geometric distance between predicted depths over the entire image sequence. The network can output depth maps with a globally consistent scale in autonomous driving scenes. Although the problem of scale ambiguity is still open, this work has laid a solid foundation for its solution.

## 3. Method

In this section, we propose a monocular absolute depth estimation method for small UAVs based on unsupervised deep learning and multi-view geometry. It takes forward-looking monocular image sequence of flight scene and UAV navigation data as input, and outputs absolute dense depth maps corresponding to the input images. The overall process is presented first, followed by details.

### 3.1. Pipeline

**Figure 3:**
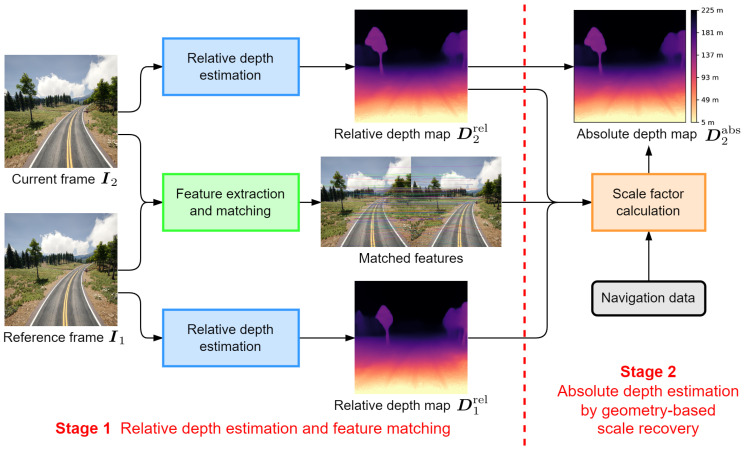
Overall process of proposed monocular absolute depth estimation method.

The overall process of our method is shown in Figure 3. For each frame in the sequence, we consider it to be the current frame $I_{2}$, and a reference frame $I_{1}$ is selected from previous frames in the sequence. The other input is the UAV navigation data, including its position and attitude at these two imaging moments. The output is a dense absolute depth map $D_{2}^{\mathrm{abs}}$ indicating the distance of each pixel in $I_{2}$. Our method can be divided into two stages.

- Stage 1, Relative depth estimation and feature matching. This stage predicts relative depth maps for reference and current frames using unsupervised deep learning. By introducing a geometry consistency loss as part of the supervision for network training, the relative depth predictions are scale-consistent under sequence. Meanwhile, feature extraction and matching is performed between these two frames.
- Stage 2, Absolute depth estimation by geometry-based scale recovery. This stage transforms the relative depth map of current frame into an absolute depth map, also known as “scale recovery”.This is achieved by calculating a scale factor between relative and absolute scales using the relative depth values of matched feature points in two frames and the UAV navigation data at these two imaging moments. In this calculation process, the real world scale is introduced by the position data, and a series of geometric transformations are performed according to its attitude angles.

### 3.2. Relative Depth Estimation and Feature Matching

#### 3.2.1. Relative Depth Estimation with Scale Consistency

**Figure 4:**
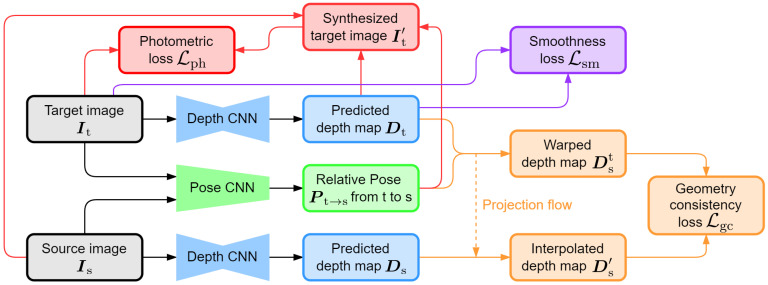
Illustration of network architecture and calculation flow of loss function.

We use the network architecture proposed by [27,28] for scale-consistent relative depth estimation. Given two consecutive images in the training sequence, the target image $I_{t}$ and the source image $I_{s}$, the goal in this subsection is to train a depth estimation network (depth CNN) to predict a dense relative depth map $D_{t}$ corresponding to $I_{t}$. The depth CNN uses an encoder-decoder structure with skip connections [29]. And ResNet-50 [30] is used as the encoder, whose weights are pre-trained on ImageNet [31]. The loss function for network training consists of three terms: photometric loss, smoothness loss and geometry consistency loss. The network architecture and the calculation flow of loss terms are illustrated in Figure 4.

Firstly, as introduced in Section 2.1, with the predicted depth map $D_{t}$ and the camera intrinsics, together with the relative pose $P_{t\to s}$ from image plane t to s predicted by the pose estimation network (pose CNN), we can reconstruct a synthesized target image $I_{t}^{\prime }$ from $I_{s}$ by the operation of inverse warping. The photometric loss $L_{\mathrm{ph}}$ is composed of the structural similarity (SSIM) [32] and the $L_{1}$ loss between $I_{t}^{\prime }$ and $I_{t}$.

(3) $$L_{\mathrm{ph}}=\frac{1}{\left|V\right|}\sum_{p\in V}\left(\frac{\eta_{s}}{2}\left(1-{\mathrm{SSIM}}_{tt^{\prime }}\left(p\right)\right)+\left(1-\eta_{s}\right)∥I_{t}\left(p\right)-I_{t}^{\prime }\left(p\right){∥}_{1}\right)$$

where *V* stands for the set of valid points *p* that can be successfully projected from $I_{t}$ to the source image plane, and |V| denotes the number of valid points. Following [27], we take $\eta_{s}=0.85$.

Secondly, the edge-aware smoothness loss $L_{\mathrm{sm}}$ is calculated from $I_{t}$ and $D_{t}$, to regularize the depth map in low texture and homogeneous regions.

(4) $$L_{\mathrm{sm}}=\sum_{p}{\left(e^{-\nabla I_{t}\left(p\right)}\cdot D_{t}\left(p\right)\right)}^{2}$$

where ∇ is the first derivative of pixel values along spatial directions.

Most importantly, the scale consistency between predicted depth maps is ensured by minimizing the differences between consecutive depth maps and then extended to the whole sequence. With $P_{t\to s}$, we warp $D_{t}$ to the source image plane, obtaining a warped depth map $D_{s}^{t}$. Then, we interpolate $D_{s}$ to align to the same pixel grid as $D_{s}^{t}$, obtaining an interpolated depth map $D_{s}^{\prime }$. The geometry consistency loss $L_{\mathrm{gc}}$ is defined as the difference between $D_{s}^{t}$ and $D_{s}^{\prime }$.

(5) $$L_{\mathrm{gc}}=\frac{1}{\left|V\right|}\sum_{p\in V}\frac{|D_{s}^{t}\left(p\right)-D_{s}^{\prime }\left(p\right)|}{D_{s}^{t}\left(p\right)+D_{s}^{\prime }\left(p\right)}$$

Finally, the overall loss function is the weighted sum of these three loss terms.

(6) $$L=\eta_{\mathrm{ph}}L_{\mathrm{ph}}+\eta_{\mathrm{sm}}L_{\mathrm{sm}}+\eta_{\mathrm{gc}}L_{\mathrm{gc}}$$

Following [27] again, we set the weights to $\eta_{\mathrm{ph}}=1.0,\;\eta_{\mathrm{sm}}=0.1,\;\eta_{\mathrm{gc}}=0.5$.

During testing, only the depth CNN is used to predict a relative depth map with the same resolution as the input RGB image from a monocular image sequence.

#### 3.2.2. Feature Extraction and Matching

With the scale consistency of relative depth maps corresponding to the image sequence, we can track the changes in relative depth values of a 3D point at different views, and then determine its absolute depth in a certain view. To achieve this, we should first find the pixel positions of this point in different frames and the correspondence. Therefore, we use the classical scale-invariant feature transform (SIFT) [33] to extract feature points from each frame and find the matching relations between them. We only give a brief description here, as this image processing method is well studied and can be easily implemented by established computer vision tools such as OpenCV.

For SIFT feature extraction, we search for SIFT key points in the gray images converted from original reference and current frames. Then, we compute a 128-dimensional SIFT descriptor for each key point.

For SIFT feature matching, we use the method of ratio test proposed in [33]. The $L_{2}$ norm between the descriptors of any two feature points in two frames is computed as the distance metric. Then, for each feature point in $I_{2}$, we compute the ratio of the distances of its best match (with minimum distance) and the second best match (with second minimum distance) in $I_{1}$. If the ratio is not greater than the maximum ratio MR, we consider this pair of feature points to be a matched pair. In this paper, we set the threshold MR=0.7.

### 3.3. Absolute Depth Estimation by Geometry-Based Scale Recovery

Inspired by [4], we try to find an intermediate quantity to establish a relation between relative and absolute scales, and calculate a scale factor from its relative and absolute values. From the perspective of UAV flight, we assign this important task to the camera’s displacement between two frames, i.e., the Euclidean distance between two camera positions where these two images are captured. It is also called “the length of baseline”, since its function is similar to the baseline between binocular cameras in stereo vision. As the camera is fixed on the UAV, the absolute value of baseline length is also the displacement of the UAV, which can be computed from the data provided by its navigation sensor. Taking GPS as an example, by converting its output data (latitude, longitude and altitude) into a geographic coordinate system, the position coordinates of the UAV can be computed, and the baseline length is the distance between the UAV positions at two imaging moments. The influence of sensor noises will be analyzed in the experiment. Then, the remaining task is to calculate the relative baseline length using the relative depth values of matched feature points in two frames, together with the attitude angles and sight angles we know.

#### 3.3.1. Calculation of Relative Baseline Length

**Figure 5:**
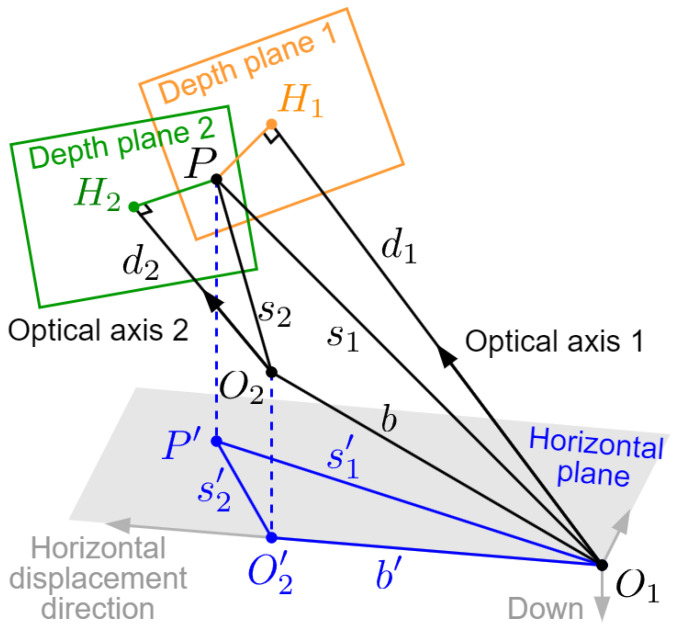
Imaging model of two frames.

The imaging model is illustrated in Figure 5. Two images in the testing sequence, reference frame $I_{1}$ and current frame $I_{2}$ (which may not be consecutive), are captured by the on-board camera at two moments. The positions of the UAV at these two moments are $O_{1}$ and $O_{2}$. For clarity, we hide image planes and show only the depth planes which pass through the 3D position of the feature point and are parallel to respective image planes. Note that the attitudes of the UAV at the two moments are different, so the two image planes are not parallel, nor are the two depth planes. For the world coordinate system, the *x*-axis points in the direction of the UAV’s horizontal displacement, the *z*-axis is vertically down and the *y*-axis follows the right-hand rule. The earth curvature is not taken into account. Correspondingly, we use front-right-down (FRD) coordinates as the UAV’s body coordinate system, and the directions of its attitude angles (three Euler angles: roll, pitch and yaw) are determined according to the right-hand rule based on the axes of body system. For simplicity, we assume that the installation position of navigation sensor, the mass center of the UAV and the optical center of camera are the same. In this way, the displacement of the UAV can be computed from the data of navigation senor, and is also consistent with the displacement of camera; thus, it can be used in the imaging model. Also, the optical axis of camera coincides with the *x*-axis (front) of the UAV’s body system. Then, the attitude angles of the UAV at these two moments can be denoted as $\left(\theta_{1},\phi_{1},\psi_{1}\right)$ and $\left(\theta_{2},\phi_{2},\psi_{2}\right)$ respectively.

There may be numerous pairs of matched feature points between two frames. But in this subsection, we focus on the imaging geometric relation of only one pair among them. The 3D position of this point is *P*. According to the pixel position of its projection on two image planes, we can find the relative depth values $d_{1}=O_{1}H_{1}$ and $d_{2}=O_{2}H_{2}$ from the corresponding depth maps. Note that the pixel value in depth maps predicted by CNN is planar depth, which represents the perpendicular distance from the optical center to the depth plane, rather than the slope distance s=OP.

The length and angle relations in Figure 5 are complicated, and the baseline length $b=O_{1}O_{2}$ cannot be directly expressed by the quantities we know. To solve this, we perform dimension reduction by projecting some key elements to the geographic horizontal plane for calculation, as shown in blue in Figure 5. The baseline $O_{1}O_{2}$ is projected as $O_{1}O_{2}^{\prime }$ (the superscript in this figure represents the projected elements), and the task is changed to express its length $b^{\prime }$ by the quantities we know. In this model, for the reference frame, the planar depth $d_{1}=O_{1}H_{1}$ and slope distance (also called “perspective depth”) $s_{1}=O_{1}P$ can be related by two sight angles $\left(\mu_{1},\nu_{1}\right)$ of point *P* relative to camera 1, which can be calculated according to the pixel position of *P* in frame 1. The horizontal projection of perspective depth, including its length $s_{1}^{\prime }=O_{1}P^{\prime }$ and direction angle, can be calculated by performing a series of coordinate transformations involving angles $\left(\psi_{1},\theta_{1}\right)$ and $\left(\mu_{1},\nu_{1}\right)$. The same applies to the current frame and $s_{2}^{\prime }$. Finally, the lengths $\left(b^{\prime },s_{1}^{\prime },s_{2}^{\prime }\right)$ can be related by the cosine law within $▵O_{1}P^{\prime }O_{2}^{\prime }$, and $b^{\prime }$ can be expressed by known quantities. The specific calculation process is as follows.

**Figure 6:**
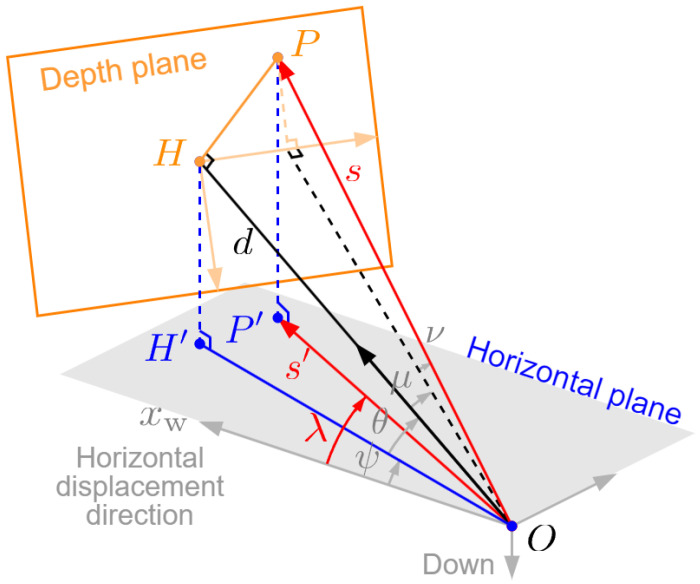
Calculating horizontal projection of perspective depth in one frame.

**Figure 7:**
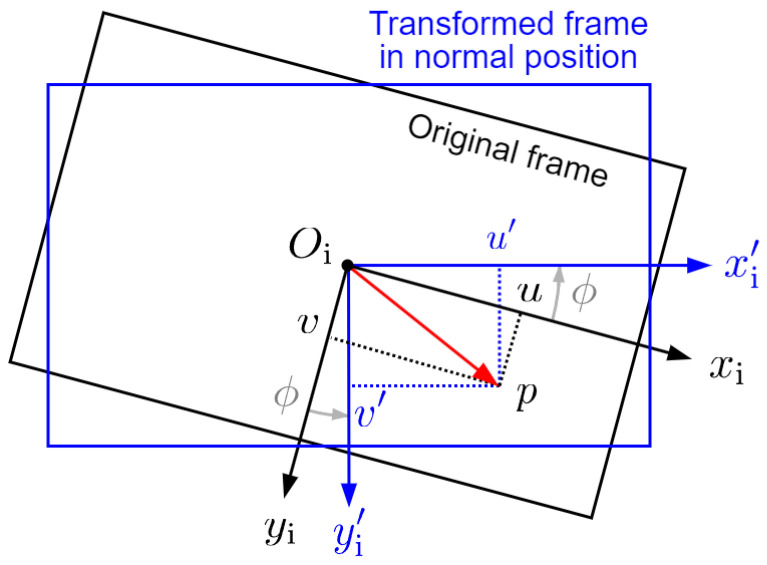
Rolling transformation in the image plane.

For each frame, we calculate the horizontal projection of perspective depth, including its length $s^{\prime }$ and direction angle $\lambda =∠x_{w}OP^{\prime }$, as shown in Figure 6 (omitting the subscripts 1 and 2 for a while). First, we compensate for the influence of the UAV’s roll angle. Since CNN predictions are planar depths, rolling does not affect depth values, but only changes the pixel position of projected point *p* in the image, and consequently, its sight angles. As shown in Figure 7, we rotate the image plane by −ϕ about the *z*-axis (perpendicular to the paper pointing inwards) of image coordinate system, and the frame is returned to its normal position in the image plane. This is equivalent to rotating the vector $\vec{O_{i}p}$ by ϕ degrees about the *z*-axis, which can be expressed by (with the third dimension omitted)

(7) $$\left[\begin{array}{c} u^{\prime }\\ v^{\prime } \end{array}\right]=\left[\begin{array}{cc} \cos\phi & -\sin\phi \\ \sin\phi & \cos\phi \end{array}\right]\left[\begin{array}{c} u\\ v \end{array}\right]$$

where ${\left(u,v\right)}^{T}$ and ${\left(u^{\prime },v^{\prime }\right)}^{T}$ are the pixel coordinates of *p* in original and transformed frames. Then, the sight angle of *p* refers to the angle between the optical axis of camera and the line connecting this point to the optical center, and can be decomposed into drift angle μ and dip angle ν along the $x_{i}$ and $y_{i}$ directions respectively, which can be calculated by

(8) $$\begin{array}{cc} \mu & =\arctan\left(\frac{2u^{\prime }}{w}\tan\frac{\alpha }{2}\right)\\ \nu & =\arctan\left(-\frac{2v^{\prime }}{h}\tan\frac{\beta }{2}\right) \end{array}$$

where w×h is the resolution of image, and α×β is camera’s field of view (FOV). Note that the positive direction of ν is upward, which is opposite to the direction of the *y*-axis of image system, so there is a negative sign in the second equation. Now that the image is rolling transformed, we only need to consider the pitch and yaw angles then.

According to the camera imaging principle, the sight angles of point *P* is the same as those of its projection *p* on the image, i.e., the angles (μ,ν). According to trigonometry in Figure 6, the perspective depth can be calculated by

(9) $$s=\frac{d}{\cos\mu \cos\nu }$$

Now we calculate its horizontal projection by applying a series of coordinate transformations. Imagine that we rotate the original world coordinate system by ψ about its *z*-axis (downward) (yawing transformation), and then by θ about the resulting *y*-axis (pitching transformation). At this point, the *x*-axis of the resulting system coincides with camera’s optical axis (along with $\vec{OH}$). Then, we rotate the resulting system by μ about its *z*-axis, and then by ν about the resulting *y*-axis (can be viewed as another pair of yawing and pitching transformations). As a result, the *x*-axis of the final system coincides with the direction of perspective depth (along with $\vec{OP}$). These operations can be expressed by a rotation matrix from the original world system to the final system as $R_{\mathrm{world}}^{\mathrm{final}}=R_{y}{\left(\nu \right)}^{-1}R_{z}{\left(\mu \right)}^{-1}R_{y}{\left(\theta \right)}^{-1}R_{z}{\left(\psi \right)}^{-1}$, where $R_{z}\left(\psi \right)$ denotes the transformation of rotating ψ about the *z*-axis, and same with the rest. Note that the rotation matrix of a vector is the inverse of the rotation matrix of the coordinate system. Now, let vector $\vec{OP}$ be expressed in the final system as $s_{\mathrm{final}}={\left(s,0,0\right)}^{T}$, and its coordinates in the original world system $s_{\mathrm{world}}={\left(s_{x},s_{y},s_{z}\right)}^{T}$ can be calculated by

(10) $$\begin{array}{cc} s_{\mathrm{world}}\; & =R_{\mathrm{final}}^{\mathrm{world}}\cdot s_{\mathrm{final}}={\left(R_{\mathrm{world}}^{\mathrm{final}}\right)}^{-1}\cdot s_{\mathrm{final}}\\ \; & ={\left(R_{y}{\left(\nu \right)}^{-1}R_{z}{\left(\mu \right)}^{-1}R_{y}{\left(\theta \right)}^{-1}R_{z}{\left(\psi \right)}^{-1}\right)}^{-1}\cdot s_{\mathrm{final}}\\ \; & =R_{z}\left(\psi \right)R_{y}\left(\theta \right)R_{z}\left(\mu \right)R_{y}\left(\nu \right)\cdot s_{\mathrm{final}}\\ & =\left[\begin{array}{ccc} \cos\psi & -\sin\psi & 0\\ \sin\psi & \cos\psi & 0\\ 0 & 0 & 1 \end{array}\right]\left[\begin{array}{ccc} \cos\theta & 0 & \sin\theta \\ 0 & 1 & 0\\ -\sin\theta & 0 & \cos\theta \end{array}\right]\\ & \;\left[\begin{array}{ccc} \cos\mu & -\sin\mu & 0\\ \sin\mu & \cos\mu & 0\\ 0 & 0 & 1 \end{array}\right]\left[\begin{array}{ccc} \cos\nu & 0 & \sin\nu \\ 0 & 1 & 0\\ -\sin\nu & 0 & \cos\nu \end{array}\right]\left[\begin{array}{c} s\\ 0\\ 0 \end{array}\right]\\ & =s\left[\begin{array}{c} \cos\nu \cos\mu \cos\theta \cos\psi -\cos\nu \sin\mu \sin\psi -\sin\nu \sin\theta \cos\psi \\ \cos\nu \cos\mu \cos\theta \sin\psi +\cos\nu \sin\mu \cos\psi -\sin\nu \sin\theta \sin\psi \\ -\cos\nu \cos\mu \sin\theta -\sin\nu \cos\theta \end{array}\right]\\ \; & \overset{\mathrm{def}}{=}{\left(s_{x},s_{y},s_{z}\right)}^{T} \end{array}$$

The horizontal projection of perspective depth vector $\vec{OP^{\prime }}$ in the world system can be expressed as $s_{\mathrm{world}}^{\prime }={\left(s_{x},s_{y},0\right)}^{T}$, whose length $s^{\prime }$ and direction angle λ are

(11) $$\begin{array}{cc} s^{\prime } & =\sqrt{s_{x}^{2}+s_{y}^{2}}\\ \lambda & =\arctan\frac{s_{y}}{s_{x}} \end{array}$$

**Figure 8:**
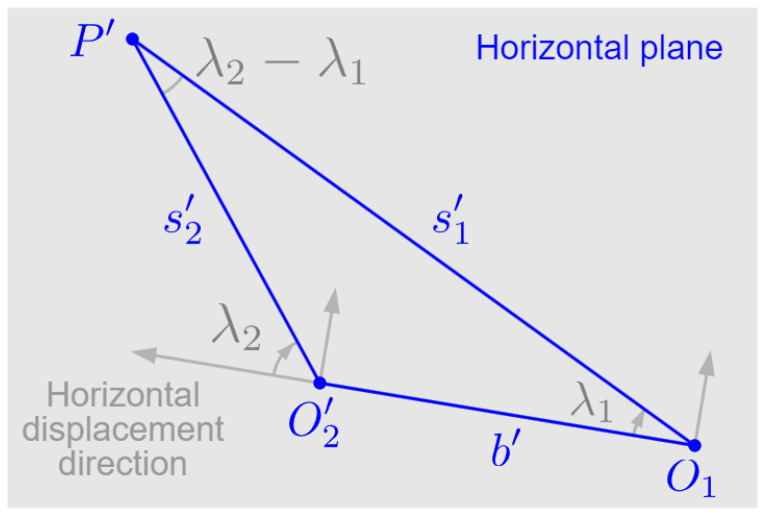
Calculating horizontal baseline length in the horizontal plane.

We perform the above operations and calculations for both two frames. And the last step is carried out in the horizontal plane, as shown in Figure 8. With the information of two horizontal projections of perspective depth, i.e., $\left(s_{1}^{\prime },\lambda_{1}\right)$ and $\left(s_{2}^{\prime },\lambda_{2}\right)$, the horizontal baseline length $b^{\prime }$ can be calculated according to the cosine law in $▵O_{1}P^{\prime }O_{2}^{\prime }$.

(12) $$b^{\prime }=\sqrt{{s_{1}^{\prime }}^{2}+{s_{2}^{\prime }}^{2}-2s_{1}^{\prime }s_{2}^{\prime }\cos\left(\lambda_{2}-\lambda_{1}\right)}$$

**Figure 9:**
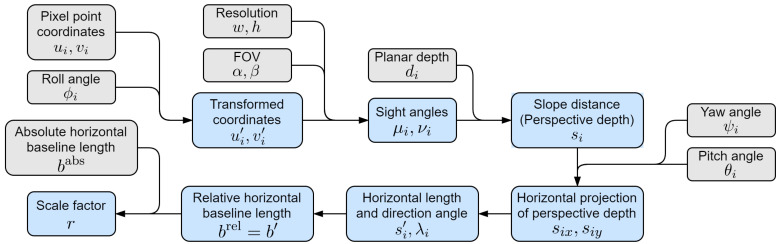
Calculation flow of relative horizontal baseline length. Gray text boxes indicate input data, and i=1,2 for reference and current frames.

The overall calculation flow is summarized in Figure 9.

#### 3.3.2. Calculation of Scale Factor

Assume that there are a total of *q* pairs of matched feature points between reference and current frames. According to our calculation process above, each pair of matched points can generate a relative value of horizontal baseline length, denoted as $b_{k}^{\mathrm{rel}}=b_{k}^{\prime }$. And accordingly, a scale factor $r_{k}$ between relative and absolute scales can be calculated by

(13) $$r_{k}=\frac{b_{k}^{\mathrm{rel}}}{b^{\mathrm{abs}}}$$

where $b^{\mathrm{abs}}$ is the horizontal displacement of the UAV between two moments with real physical unit, which can be computed from the UAV positions.

These feature points are scattered throughout the images. Therefore, to make better use of them, we take the median value of the set of $r_{k}$ as the global scale factor *r*.

(14) $$r=\underset{k=1,\cdots ,q}{\mathrm{median}}\left\{r_{k}\right\}=\underset{k=1,\cdots ,q}{\mathrm{median}}\left\{\frac{b_{k}^{\mathrm{rel}}}{b^{\mathrm{abs}}}\right\}$$

The operation of taking the median not only mitigates the negative effect of mismatches during feature matching, but also provides a reasonable global scale for the scene.

Since the global scale factor is calculated from the information of the reference and current frames, it is applicable to both frames. On the other hand, each frame in a sequence (except several frames at the beginning and end) can be used twice, once as the current frame and once as the reference, thus obtaining two associated global scale factors. In our method, in order to make full use of the images and improve accuracy, the final scale factor of a certain frame is taken as the average of its two associated global scale factors. Finally, the scale recovery of this frame is completed by multiplying the relative depth map $D^{\mathrm{rel}}$ by the final scale factor pixel by pixel, resulting in an absolute dense depth map $D^{\mathrm{abs}}$.

(15) $$D^{\mathrm{abs}}=\frac{r^{\mathrm{cur}}+r^{\mathrm{ref}}}{2}\cdot D^{\mathrm{rel}}$$

where $r_{\mathrm{cur}}$ and $r_{\mathrm{ref}}$ are the global scale factors when the frame is used as the current and the reference for calculation respectively.

## 4. Experiment

In this section, we verify the effectiveness of our monocular absolute depth estimation method in UAV flight scenes. We introduce the public dataset we use, and describe the common metrics for depth estimation. After providing implementation details, we present quantitative and qualitative results and analyses to demonstrate the effectiveness of our method and to show its advantages and weaknesses.

### 4.1. Dataset

In contrast to autonomous driving applications, datasets and tools for outdoor aerial depth estimation are relatively rare. One important reason is that collecting GT depth data during actual flights is a costly endeavor. For this reason, the simulation plugin AirSim [34] built on the graphics engine Unreal Engine has become very popular, as it allows the acquisition of image sequences and corresponding dense GT depth maps, as well as precise UAV navigation data, using only a high-performance graphic workstation.

We choose a public aerial image dataset called “Mid-Air” [16] for the experiment. It is a large synthetic dataset constructed by AirSim for low-altitude UAV flights in unstructured natural environments. Data from multiple sensors and different types of GTs are available, including stereo RGB images, depth and disparity maps, and semantic segmentation, all at a resolution of 1024×1024 pixels and a field of view (FOV) of 90°. Notably, all the 54 trajectories in this dataset contain images taken in different climates and weather conditions.

**Figure 10:**
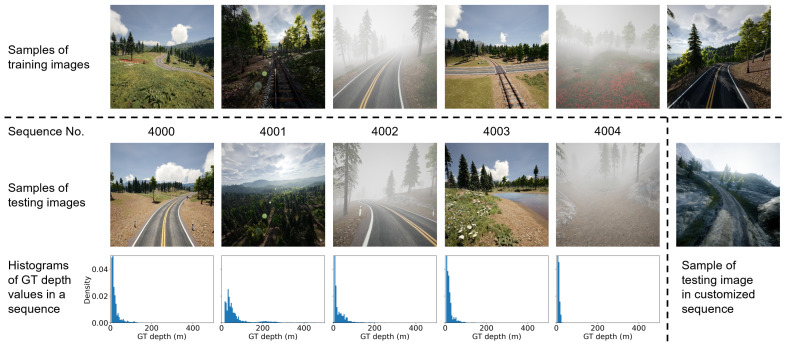
Image samples in the training and testing sequences of PLE environment of Mid-Air dataset and our customized testing sequence.

In this paper, we choose the 24 trajectories in the “PLE” environment of Mid-Air dataset [16] to train the relative depth estimation network. And we choose 5 trajectories in the same environment from its official benchmark to test and evaluate our absolute depth estimation method. For simplicity, we only use the sequences synthesized in the spring season condition for training and testing, but they include four weather conditions of sunny, sunset, foggy and cloudy. Some samples of training and testing images are shown in Figure 10. Below the image samples of each testing sequence, a frequency distribution histogram of the GT depth values belonging to the ground area in the sequence is provided, representing the depth variation of the scene. The values in the GT depth maps have a long-tailed distribution. The more concentrated the data in the histogram is near the vertical axis, the flatter the depth variation in the scene.

In addition to using existing datasets, we customize a flight trajectory in an open-source virtual environment of AirSim [34] called “Landscape Mountains” to generate image sequences at different speeds, which is used in Section 4.4.4 (c). We generate 845 images for a constant speed sequence and 856 images for a variable speed sequence, with the same resolution and FOV as Mid-Air [16]. The image sample is shown in the lower right corner of Figure 10.

### 4.2. Implementation Details

The training method of pre-training on large-scale datasets and then fine-tuning with the required scene data has been proven to be able to improve the performance of depth estimation models [13,14,27]. Therefore, we take the depth estimation model provided by SC-Depth [28] (the ResNet-50 version) as the pre-trained model, which is trained on the large-scale road driving image dataset KITTI [35]. We then perform fine-tuning on the Mid-Air dataset. When preparing the data, we sample the Mid-Air image sequence every 2 frames, in order to match the displacement between adjacent frames with KITTI, so that the disparity between frames is also at the same level for these two datasets, which can be beneficial for transfer learning. After sampling, there are a total of 26,466 training images and 815 testing images, with an average frame displacement of 0.9 m.

We divide 10% of continuous images from each training sequence as the validation set. Then, we train the relative depth estimation network on the remaining training samples for 15 epochs using the Adam optimization method [36], and validate the model at the end of each epoch. During training, we set the batch size to 8 and resize the images to a resolution of 256×256 pixels. Other hyper-parameters are the same as in [28], including a learning rate of $1\times 10^{-4}$ with no decay. The training process is implemented using PyTorch [37] v1.8.0, and it takes about 16 h on the NVIDIA GeForce RTX 3060 12 GB GPU device with Windows 11.

For absolute depth estimation, we ignore the sky area as its GT depth should be infinite. By excluding the pixels whose GT depth value is greater than 10 km, both the feature engineering and the scale recovery process are performed only on the pixels belonging to the ground area. We also exclude a thin strip of pixels on the four sides of image, as most depth estimation works since [17] have done. The interval between reference and current frames is 1, i.e., the inputs to scale factor calculation are consecutive frames. The performance with different intervals will be discussed later in Section 4.4.4.

In addition to using the GT values of navigation data to estimate absolute depth, we also use the sensor measurements of GPS and IMU provided in the Mid-Air dataset [16], both of which simulate the noise. The GPS data contain position measurements of UAVs, and its error comes from the additive noise on pseudoranges. We interpolate the raw data of GPS positions from the rate of 1 Hz to 12.5 Hz to synchronize with the image stream, and treat it as the noisy position data. The IMU data contain 3-axis angular velocity measurements under the UAV body coordinate system, which are measured by the gyroscope. Its errors come from the bias (manifested as a Gaussian random walk process with drift) and the measurement noise (following a normal distribution with a mean of zero). We convert the angular velocity measurements to the same geographic coordinate system as the attitude angles, and then generate the noisy attitude data by integration.

### 4.3. Metrics

The commonly used metrics for depth estimation task include errors and accuracies [17]. Error metrics are absolute relative error (Abs Rel, i.e., the mean relative error (MRE)), square relative error (Sq Rel), root mean square error (RMSE) and logarithmic RMSE (RMSE log), the lower the better. And accuracy metrics are the percentages of pixels that meet a series of thresholds, the higher the better. The metrics are defined as follows. And all the data presented in this section are the averages of these metrics across all testing images.

(16) $$\begin{array}{cc} \mathrm{Abs}\;\mathrm{Rel}: & \;\frac{1}{T}\sum_{i=1}^{T}\frac{|d_{i}-d_{i}^{*}|}{d_{i}^{*}} \end{array}$$

(17) $$\begin{array}{cc} \mathrm{Sq}\;\mathrm{Rel}: & \;\frac{1}{T}\sum_{i=1}^{T}\frac{∥d_{i}-d_{i}^{*}{∥}^{2}}{d_{i}^{*}} \end{array}$$

(18) $$\begin{array}{cc} \mathrm{RMSE}: & \;\sqrt{\frac{1}{T}\sum_{i=1}^{T}{∥d_{i}-d_{i}^{*}∥}^{2}} \end{array}$$

(19) $$\begin{array}{cc} \mathrm{RMSE}\;\log: & \;\sqrt{\frac{1}{T}\sum_{i=1}^{T}{∥\log d_{i}-\log d_{i}^{*}∥}^{2}} \end{array}$$

(20) $$\begin{array}{cc} \mathrm{Accuracies}: & \;\%\;\mathrm{of}\;d_{i}\;s.t.\max\left(\frac{d_{i}}{d_{i}^{*}},\frac{d_{i}^{*}}{d_{i}}\right)=\delta <\mathrm{thr} \end{array}$$

where $d_{i}$ and $d_{i}^{*}$ are the predicted and GT absolute depth value at the *i*-th pixel respectively, *T* is the number of pixels for evaluation in an image and $\mathrm{thr}=1.25^{t}$ is the accuracy threshold where t=1,2,3.

For relative depth maps, the common evaluation mode is called “median scaling (MS)”. The ratio of the median of the GT depth map to the median of the relative depth map is calculated as the scale factor, which is then used to scale the relative depth map by pixel-wise multiplication. Metrics are computed between the scaled prediction and GT. However, for absolute depth maps, metrics can be computed directly between prediction and GT, which is represented as “direct” in the tables below. In general, we select the pixels with GT depth values less than 80 m for evaluation. And the performance with different depth ranges will also be discussed later in Section 4.4.4.

### 4.4. Quantitative Results

#### 4.4.1. Performance

To demonstrate the advantage of absolute depth estimation results over relative depth estimation, the metrics of relative and absolute depth estimation on the testing sequences of Mid-Air [16] are shown in Table 1.

As shown in Table 1, we use the SC-Depth [28] model trained on KITTI for comparison, whose predictions are relative (row 1). By fine-tuning on Mid-Air, there is a noticeable improvement in the results (row 2). However, if median scaling is not used in evaluation, the metrics can be quite poor due to scale ambiguity (row 3). Most practical applications cannot provide GT data (even sparse GT), so median scaling is not feasible, which limits the value of relative depth maps. After scale recovery, the error of absolute depth maps generated by our method is about 29.5% in Abs Rel (row 4). This is still promising because these results can be directly used for other tasks such as vehicle guidance and control, although there is a 10% performance degradation in the scale recovery process from relative to absolute results. According to our understanding, this could be attributed to the feature matching error.

In practice, UAV navigation data are generated from two commonly used navigation sensors, namely GPS and IMU, which are usually noisy. To demonstrate the effectiveness of our method in the presence of sensor noise, we take the noisy position and attitude data generated from sensor measurements as the input to the scale recovery stage and compare the results with those using GT data. The metrics are shown in Table 2.

As shown in Table 2, the performance of absolute depth estimation degrades when there is noise in the navigation data. When only the position data provided by GPS are noisy, the metric Abs Rel degrades by about 1%. When only the attitude data generated from IMU are noisy, this metric degrades by about 0.5%. If both are noisy, the degradation adds up, but only by 1.5%, and the depth estimation results are still useful. Therefore, our method is robust to navigation sensor noise. The reason is that the noise in GPS measurements is additive, and the baseline length used in our method is obtained by subtracting the position data at two moments, so that the noise with the same direction can be compensated. For IMU measurements, after converting them to the attitude data, we found that the magnitude of noise is small, since the cumulative error at the end of a sequence is less than $5^{\circ }$. On the other hand, as shown in Figure 9, the scale recovery process involves many intermediate steps. The noise of attitude angles will be diluted by other quantities with larger errors, such as relative depths and sight angles associated with feature matching.

Combining the results of Table 1 and Table 2, it can be seen that out of the 31% error in Abs Rel of the absolute depth estimation results, 19% comes from relative depth estimation, 10.5% is due to the feature matching error and the remaining 1.5% is related to sensor noise. Therefore, the main factor affecting the performance of our method is the accuracy of vision algorithms, while the effect of navigation sensor noise is not as significant.

#### 4.4.2. Comparison with Other Methods

Our method is oriented towards flight scenes, but in order to compare with the results reported by other related methods, we make a comparison on the KITTI road driving image dataset [35], which is currently the largest benchmark for MDE studies. By convention, we use the Eigen split [17] with more than 600 images distributed in 28 testing sequences of KITTI for evaluation. All the methods for comparison are trained and tested only on KITTI. For each testing sequence, we apply our method to the whole sequence, and then extract the metrics of specific testing images. The results are shown in Table 3.

As shown in Table 3, it can be seen that in road driving scenes, the absolute depth estimation methods which exploiting scale information from camera height (DNet [4] and SD-SSMDE [24]) and IMU sensor (DynaDepth [25]) perform as well as the relative depth estimation results with median scaling. Their results are better than those of the methods using vehicle position for scale recovery (Pinard et al. [26] and ours). However, as mentioned in Section 2.2, the camera height is a strong geometric constraint that does not exist in other scenes such as UAV flight scenes, while the IMU and vehicle position data can be more accessible in different kinds of vehicles and scenes. Different from DynaDepth [25], our method does not extract absolute scale from IMU, nor does it require IMU data to be included in the training dataset, but only obtains the attitude angles from it during scale recovery. Therefore, the architecture is simpler because our network is only used for relative depth estimation, and the scale recovery algorithm explicitly serves as a post-processing stage for relative depth results based on multi-view geometry, rather than integrating multiple sensors into a sophisticated architecture as in [25]. Finally, our method outperforms the method proposed by Pinard et al. [26] in more than half of the metrics, with a smaller relative error (indicated by Abs Rel and Sq Rel) and fewer outliers (indicated by RMSE and RMSE log). Unlike [26], our method does not require the vehicle speed to be constant and known, and thus has a wider range of applications.

#### 4.4.3. Ablation Study

We validate the effectiveness of our geometry-based scale recovery algorithm through an ablation study. In the scale recovery stage, we prohibit the use of attitude angles, but calculate the scale factor only from images and the horizontal baseline length, which is equivalent to assuming that the vehicle is moving in a horizontal straight line. Metrics are also calculated in the Mid-Air and KITTI testing sets and are shown in Table 4.

As shown in Table 4, without using attitude angles for geometric transformation, the performance is degraded in all the metrics on both two datasets, which validates the effectiveness of the proposed geometry-based scale recovery algorithm. This is because both the rotation compensation of the image frame based on the roll angle and the horizontal projection of perspective depth based on the pitch and yaw angles ensure that the scale factor calculation process is correct and accurate. And for KITTI, although there are many straight sections in road driving scenes, the orientation of vehicle often changes dramatically during cornering. This demonstrates the need to introduce attitude information for absolute depth estimation both in the air and on the ground.

#### 4.4.4. Results with Different Settings

(a) Frame intervals

The baseline length is determined by the frame interval between reference and current frames. In stereo vision, it is generally accepted that a longer baseline between binocular cameras contributes to a larger range of perception. In monocular image sequences, increasing the distance between successive images may have the same effect as extending the baseline. However, this does not appear to be completely beneficial for absolute MDE.

**Figure 11:**
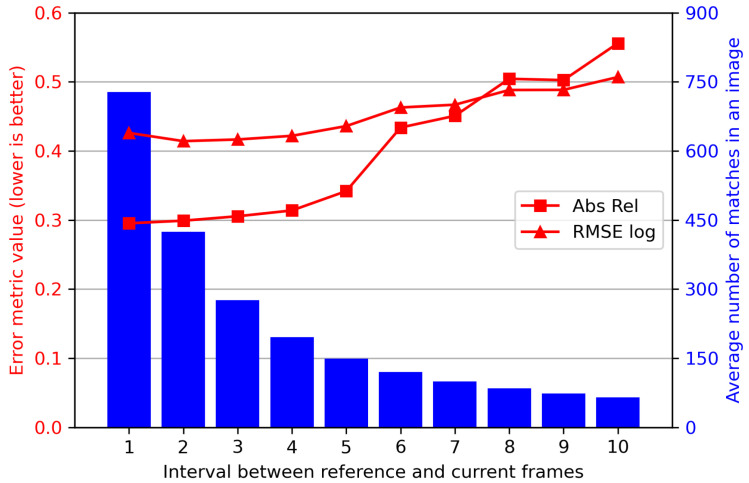
Evaluation metrics and average number of matched features with different intervals between reference and current frames.

Figure 11 shows the absolute depth estimation metrics Abs Rel and RMSE log with different intervals between reference and current frames, as well as the average number of matched feature points per image. The overall trend is that, the smaller the frame interval, i.e., the closer the two frames used to calculate the scale factor, the better the absolute depth estimation result. One reason for this is that closer frames have a higher image similarity and more matched features, as shown by the blue bars in the figure. Since that our method is based on feature correspondence between images, the calculated scale factor tends to be more accurate with more matched pairs. However, it should be noted that the distance between two images cannot be reduced infinitely, as estimating the depth of pixels requires a clear disparity between two images.

(b) Depth ranges and variations

The depth in road driving scenes is typically within 200 m, but in UAV flight scenes, the depth can exceed 1000 m, along with significant variation. However, not all the flight image sequences have such a large range. In some cases, the depth is still concentrated within a few hundred meters, with a relatively minor variation. The range and variation of scene depth can affect the performance of absolute depth estimation.

**Figure 12:**
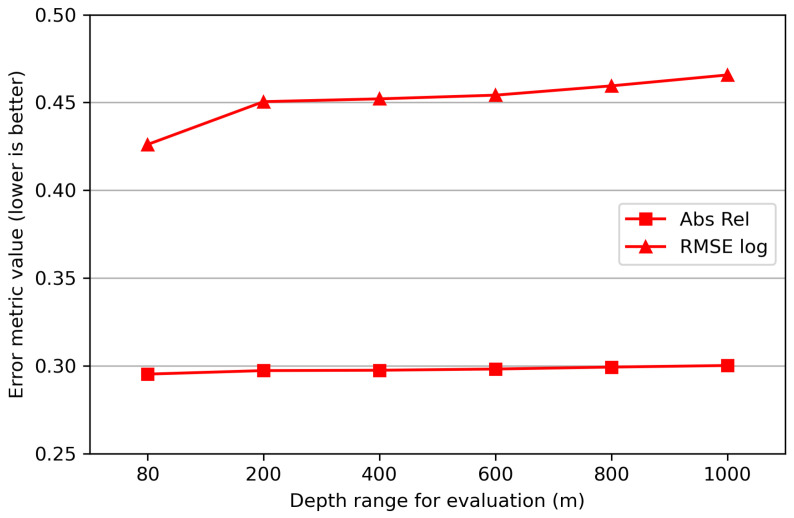
Evaluation metrics with different depth ranges.

Figure 12 shows the absolute depth estimation metrics Abs Rel and RMSE log with different depth ranges. Metrics are computed only from the pixels whose GT depth is within the depth range (e.g., 0–80 m). From the figure, it can be seen that the depth results of distant pixels are slightly worse than those of closer ones. It has been proved that the uncertainty of predicted depth values increases with GT depth values [19]. But we believe that this kind of uncertainty will not have a serious impact on absolute depth estimation in flight scenes, since the deterioration of metrics at long distances is not significant (less than 1% in Abs Rel from 80 to 1000 m).

For the entire testing set, there is no significant differences in the metrics across different depth ranges. However, the differences between testing sequences are more obvious because they have different depth variations. For the 5 testing sequences in the Mid-Air dataset we use, the scene depth variation can be represented by the frequency distribution histogram of GT depth values in Figure 10. For example, when comparing sequences 4000 and 4001, the former has a depth concentration of less than 200 m and a relatively smooth change, while the latter covers hundreds of meters and has a significant change. Sorting these sequences from small depth variation to large variation results in is 4004, 4000, 4003, 4002 and 4001. Table 5 shows the absolute depth estimation metrics of each sequence, by selecting the pixels with GT depth values less than 500 m for evaluation. It can be seen that our method performs better on sequences with less depth variation. The reason is that the absolute depth map is based on relative depth, while the relative depth estimation model is transferred from road driving scenes, making it good at predicting scene depth with small variation. Overall, our method is effective for UAV flight scenes with different depth ranges and variations.

(c) UAV motion

The difficulty in performing depth estimation in flight scenes is that the motion state of the UAV changes rapidly, with varying velocity and acceleration. Therefore, we analyze the effect of the magnitude of these two variables on the performance of our method.

**Figure 13:**
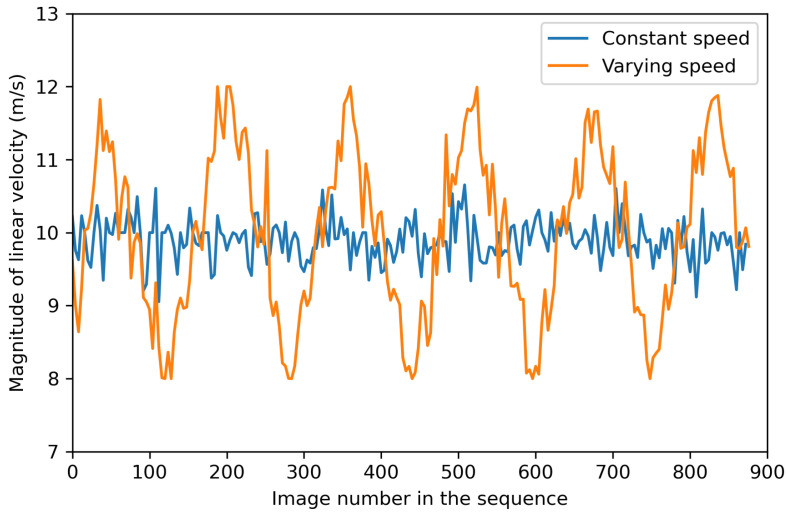
UAV speed of two customized testing image sequences.

To evaluate our method under varying speeds, we collect two image sequences at constant and varying speeds respectively under the same customized flight trajectory described in Section 4.1, with the same sampling frequency and image properties. The first sequence maintains a speed of 10 m/s, while the second varies between 8 and 12 m/s, as shown in Figure 13. Table 6 shows the absolute depth estimation metrics of these two sequences. It can be seen that the accuracy of absolute depth estimation decreases slightly by about 0.4% in Abs Rel as the speed varies.

An increase in lateral acceleration often indicates a change in motion state. For example, tilting is often accompanied by a change in flight speed, which in turn changes the scene structure in images. Thanks to the accurate GT values of linear acceleration recorded in the Mid-Air [16] dataset, we divided all the testing images into two halves based on the median of the magnitudes of UAV lateral accelerations at the imaging moments (0.92 $m/s^{2}$ in the experiment). To evaluate our method under different acceleration conditions, we compare the average absolute depth estimation metrics of the half of the images with higher lateral acceleration with the lower half. The results are shown in Table 7. It can be seen that our method performs better when the UAV has a lower magnitude of lateral acceleration, with an advantage of 2% in Abs Rel over the other half.

The above results indicate that the accuracy of absolute depth estimation will decrease as the UAV flies dynamically, which is predictable. On the one hand, changes in scene structure and motion blur can both affect the performance of vision algorithms. On the other hand, the position and attitude information of the UAV is more likely to be inaccurate and out of sync with the image stream compared to a stable flight. However, the performance degradation caused by UAV motion is acceptable, and our method is still applicable in these cases.

#### 4.4.5. Time Results

The runtime of each step of our method is recorded during the experiment, as shown in Table 8. All data are averaged over 5 repetitions. It can be seen that the relative depth estimation takes the least time since the inference is performed on a desktop GPU with CUDA acceleration. In contrast, feature matching and scale recovery take relatively longer. Furthermore, we find that the time consumption of these two steps will be reduced if the input images are downsized or the features in the image are not rich (e.g., underexposed or with large areas of low texture). However, the reduction of matched feature points may lead to a decrease in the accuracy of absolute depth estimation, which requires a trade-off based on specific applications. Our method achieves a running speed of 238.0 ms (4.2 fps) under the experimental conditions of this paper, but there is still potential for improvement so that it can be applied to embedded devices for real-time applications.

### 4.5. Qualitative Results

**Figure 14:**
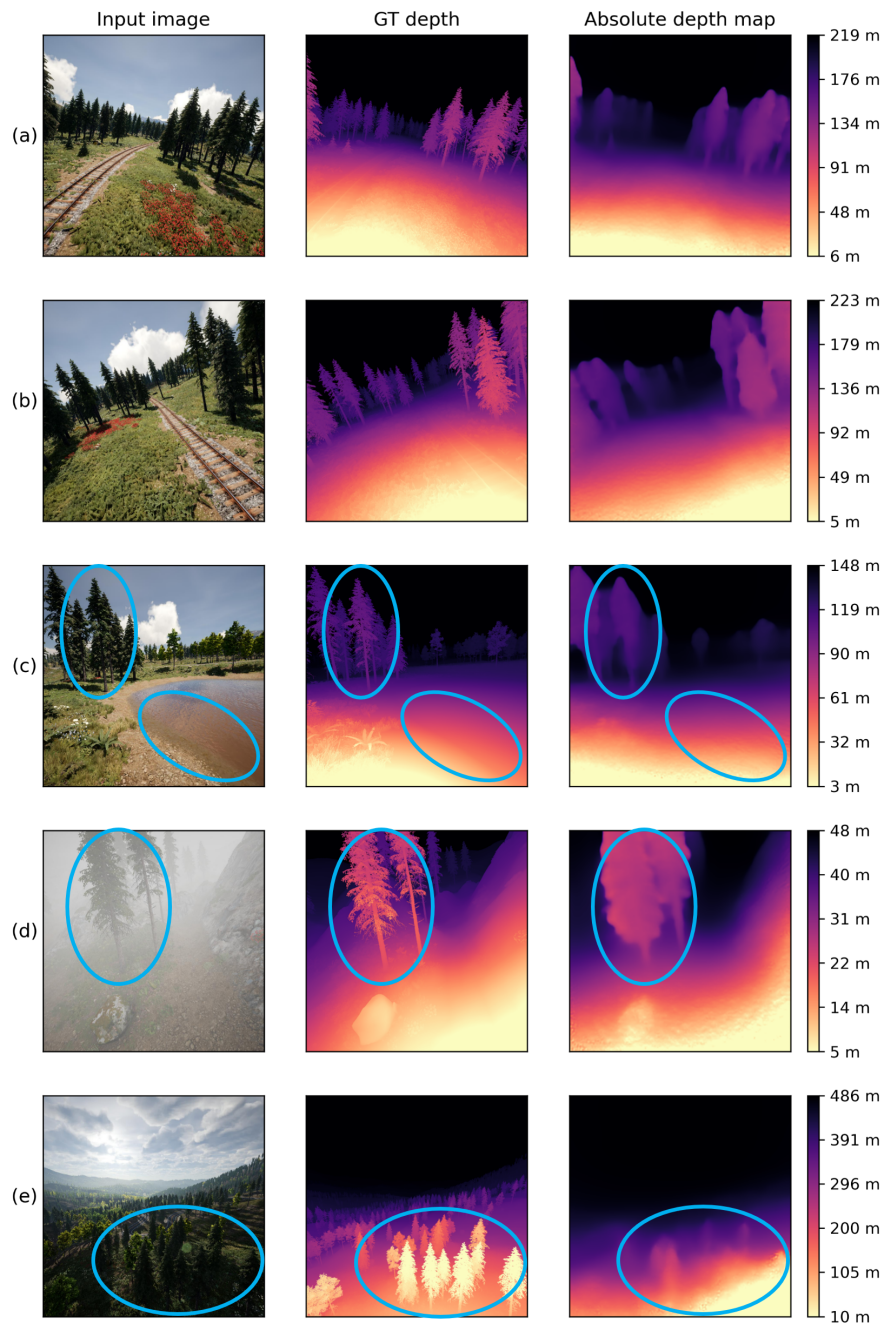
Qualitative results of our absolute depth estimation method on our flight image dataset. The areas of concern are circled in blue.

Some qualitative results of our method on the testing images of Mid-Air dataset are shown in Figure 14. The visualized depth maps is drawn by a normalization process between the maximum and minimum depth values [13], where bright parts represent near and dark parts represent far. To show the uniqueness of our absolute depth estimation method to other relative MDE methods, a color bar with absolute depth values is added to the right of each depth map.

As shown in Figure 14, examples (a) and (b) are both images taken at a time when the UAV has an obvious bank angle. And our method can provide a stable result on them, showing that it is applicable to flight scenes with changing attitude. However, there are still some limitations. First is a common problem with the CNN-based MDE methods, which is the inability to accurately estimate the depth of non-Lambertian surfaces, such as water surfaces with specular reflection in the lower right part of example (c). In addition, it does not perform well in predicting the tiny edges of objects, resulting in some artifacts, as shown by the trees in examples (c) and (d). Lastly, the results are not good in foggy and low-illumination scenes (examples (d) and (e)), where the image features are not clear enough.

## 5. Conclusions

This paper proposes a monocular absolute depth estimation method for small UAVs. The main problem addressed in this paper is to generate absolute depth maps based on relative estimations predicted by unsupervised deep learning methods and to make this process applicable to flight scenes. For this purpose, a geometry-based scale recovery algorithm is proposed. By exploiting the feature correspondence between successive images and UAV positions, the scale factor between relative and absolute scales is calculated. A series of geometric transformations based on UAV attitude angles are included to make the calculation process reasonable in flight scenes. Experiments are mainly conducted on the publicly available synthesized aerial image dataset, and the results demonstrate the effectiveness of our method in flight scenes. The main advantages of our method are as follows:

- Our method only requires the UAV to be equipped with a camera and ordinary navigation sensors, without any additional conditions or prior information, making it suitable for most small flying platforms.
- Since the scale recovery algorithm serves as a post-processing stage of relative depth estimation, it can be transplanted to various unsupervised MDE methods, which is flexible for practical applications.
- It is robust to the navigation sensor noise, and also applicable to different depth ranges and variations as well as different UAV motions.

The absolute depth information obtained by our method can be directly used for downstream tasks or applications of small UAVs, such as SLAM and obstacle avoidance path planning. Finally, the potential future work is summarized as follows:

- Reducing the relative depth estimation error and feature matching error is the key to improving the accuracy of our absolute depth estimation method. The performance could also be enhanced with the help of other sensors or visual tasks.
- Integrating feature matching and scale recovery algorithms into the CNN to achieve an end-to-end architecture for absolute depth estimation can speed up the inference, which may enable our method to operate on embedded devices for real-time applications.
- Improving the robustness of this method to different climate and weather conditions is also a worthwhile research direction.

## Figures and Tables

**Table 1:** Evaluation metrics of relative and absolute depth estimation on the Mid-Air dataset.

Method	Training Dataset	Testing Dataset	Depth Type	Evaluation Mode	Error Metrics (Lower Is Better)				Accuracy Metrics (Higher Is Better)		
					Abs Rel	Sq Rel	RMSE	RMSE log	a1	a2	a3
SC-Depth	K ^1^	MA	Relative	MS ^2^	0.2303	3.0121	9.8578	0.3491	0.6292	0.8537	0.9360
SC-Depth	K+MA	MA	Relative	MS	0.1906	1.8670	7.3232	0.2587	0.7092	0.8996	0.9631
SC-Depth	K+MA	MA	Relative	Direct	0.9404	18.0870	22.7413	2.9993	0.0000	0.0000	0.0000
Ours	K+MA	MA	Absolute	Direct	0.2953	3.2969	9.5658	0.4261	0.4069	0.6941	0.8442

^1^ “K” means the KITTI dataset, “MA” means the Mid-Air dataset, “K+MA” means pre-training on KITTI and fine-tuning on Mid-Air, same in the tables below. ^2^ “MS” means median scaling.

**Table 2:** Evaluation metrics of absolute depth estimation with noisy navigation data inputs.

Testing Dataset	Depth Type	Source of Navigation Data		Error Metrics (Lower Is Better)				Accuracy Metrics (Higher Is Better)		
		Position	Attitude	Abs Rel	Sq Rel	RMSE	RMSE log	a1	a2	a3
MA	Absolute	GT	GT	0.2953	3.2969	9.5658	0.4261	0.4069	0.6941	0.8442
MA	Absolute	GPS	GT	0.3052	3.3454	9.6172	0.4336	0.3874	0.6795	0.8408
MA	Absolute	GT	IMU	0.3000	3.2852	9.5836	0.4311	0.3844	0.6863	0.8451
MA	Absolute	GPS	IMU	0.3094	3.3360	9.6263	0.4387	0.3695	0.6670	0.8399

**Table 3:** Evaluation metrics of relative and absolute depth estimation on the KITTI dataset (the bold indicate the best).

Method	Depth Type	Source of Scale Information	Error Metrics (Lower Is Better)				Accuracy Metrics (Higher Is Better)		
			Abs Rel	Sq Rel	RMSE	RMSE log	a1	a2	a3
SC-Depth [28]	Relative	/	0.1140	0.8130	4.7060	0.1910	0.8730	0.9600	0.9820
DNet [4]	Absolute	Camera height	0.1130	0.8640	4.8120	0.1910	0.8770	0.9600	0.9810
SD-SSMDE [24]	Absolute	Camera height	0.1000	0.6610	4.2640	0.1720	0.8960	0.9670	0.9850
DynaDepth [25]	Absolute	IMU	0.1090	0.7870	4.7050	0.1950	0.8690	0.9580	0.9810
Pinard et al. [26]	Absolute	Vehicle position	0.3124	5.0302	8.4985	**0.4095**	**0.5919**	**0.7961**	0.8821
Ours	Absolute	Vehicle position	**0.3060**	**2.6086**	**7.4261**	0.6470	0.4891	0.7312	**0.8830**

**Table 4:** Evaluation metrics of ablation study on geometry-based scale recovery.

Testing Dataset	Scale Recovery Inputs	Error Metrics (Lower Is Better)				Accuracy Metrics (Higher Is Better)		
		Abs Rel	Sq Rel	RMSE	RMSE log	a1	a2	a3
MA	Image + baseline	0.3130	4.3951	10.6195	0.4927	0.4179	0.6707	0.7956
	Image + baseline + attitudes	0.2953	3.2969	9.5658	0.4261	0.4069	0.6941	0.8442
K	Image + baseline	0.4198	4.0908	9.3903	1.2373	0.2894	0.5347	0.6703
	Image + baseline + attitudes	0.3060	2.6086	7.4261	0.6470	0.4891	0.7312	0.8830

**Table 5:** Evaluation metrics of absolute depth estimation of each testing sequence in Mid-Air.

Sequence Number	Depth Range for Evaluation	Depth Variation Ranking ^1^	Error Metrics (Lower Is Better)				Accuracy Metrics (Higher Is Better)		
			Abs Rel	Sq Rel	RMSE	RMSE log	a1	a2	a3
4000	0–500 m	2	0.2453	2.6401	14.9685	0.3501	0.4249	0.7927	0.9507
4001	0–500 m	5	0.4159	15.4110	49.5378	0.6622	0.1692	0.3751	0.6125
4002	0–500 m	4	0.3538	6.2921	22.8000	0.5591	0.4248	0.6788	0.8244
4003	0–500 m	3	0.2948	3.4807	16.6903	0.4536	0.3633	0.6791	0.8316
4004	0–500 m	1	0.1800	0.4894	2.2918	0.2406	0.6327	0.9304	0.9905

^1^ Sorted in ascending order, with bigger number indicating larger depth variation.

**Table 6:** Evaluation metrics of absolute depth estimation of customized testing sequences with different speeds.

Testing Sequence	Speed Variation	Speed Value	Error Metrics (Lower Is Better)				Accuracy Metrics (Higher Is Better)		
			Abs Rel	Sq Rel	RMSE	RMSE log	a1	a2	a3
Customized	Constant	10 m/s	0.2798	3.3815	9.4691	0.3897	0.5073	0.7718	0.8844
Customized	Varying	8–12 m/s	0.2840	3.4040	9.5028	0.3878	0.4898	0.7721	0.8919

**Table 7:** Evaluation metrics of absolute depth estimation of Mid-Air testing sequences with different lateral accelerations.

Testing Dataset	Magnitude of Lateral Acceleration	Error Metrics (Lower Is Better)				Accuracy Metrics (Higher Is Better)		
		Abs Rel	Sq Rel	RMSE	RMSE log	a1	a2	a3
MA	The higher half	0.3052	3.8611	10.7055	0.4477	0.3911	0.6694	0.8293
MA	The lower half	0.2853	2.7314	8.4232	0.4045	0.4227	0.7189	0.8592

**Table 8:** Elapsed time of each step of our method.

Step	Time per Frame (ms)
Relative depth estimation	28.0
Feature matching	112.3
Scale recovery	97.7
Total	238.0

## Data Availability

Publicly available datasets are analyzed in this study. These data can be found here: The KITTI dataset at https://www.cvlibs.net/datasets/kitti/ accessed on 25 February 2021, and the Mid-Air dataset at https://midair.ulg.ac.be/ accessed on 22 October 2019.

## Abbreviations

The following abbreviations are used in this manuscript:

UAV	Unmanned aerial vehicle
MDE	Monocular depth estimation
CNN	Convolutional neural network
GT	Ground truth
DoF	Degrees of freedom
GPS	Global Positioning System
IMU	Inertial measurement unit
EKF	Extended Kalman filter
SLAM	Simultaneous localization and mapping
SSIM	Structural similarity
SIFT	Scale-invariant feature transform
FOV	Field of view
Abs Rel	Absolute relative error
MRE	Mean relative error
Sq Rel	Square relative error
RMSE	Root mean square error
RMSE log	Logarithmic root mean square error
